# Transcriptome profiling reveals male- and female-specific gene expression pattern and novel gene candidates for the control of sex determination and gonad development in *Xenopus laevis*

**DOI:** 10.1007/s00427-019-00630-y

**Published:** 2019-04-10

**Authors:** Rafal P. Piprek, Milena Damulewicz, Jean-Pierre Tassan, Malgorzata Kloc, Jacek Z. Kubiak

**Affiliations:** 1 Department of Comparative Anatomy, Institute of Zoology and Biomedical Research, Jagiellonian University, Gronostajowa 9, 30-387 Krakow, Poland; 2 Department of Cell Biology and Imaging, Institute of Zoology and Biomedical Research, Jagiellonian University, Krakow, Poland; 3 Univ Rennes, UMR 6290, Cell Cycle Group, Faculty of Medicine, Institute of Genetics and Development of Rennes, F-35000 Rennes, France; 4 The Houston Methodist Research Institute, Houston, TX USA; 5 Department of Surgery, The Houston Methodist Hospital, Houston, TX USA; 6 MD Anderson Cancer Center, University of Texas, Houston, TX USA; 7 Laboratory of Regenerative Medicine and Cell Biology, Military Institute of Hygiene and Epidemiology (WIHE), Warsaw, Poland

**Keywords:** Testis, Ovary, Sex determination, Gonad development, *Xenopus*, Transcriptome

## Abstract

*Xenopus laevis* is an amphibian (frog) species widely used in developmental biology and genetics. To unravel the molecular machinery regulating sex differentiation of *Xenopus* gonads, we analyzed for the first time the transcriptome of developing amphibian gonads covering sex determination period. We applied microarray at four developmental stages: (i) NF50 (undifferentiated gonad during sex determination), (ii) NF53 (the onset of sexual differentiation of the gonads), (iii) NF56 (sexual differentiation of the gonads), and (iv) NF62 (developmental progression of differentiated gonads). Our analysis showed that during the NF50, the genetic female (ZW) gonads expressed more sex-specific genes than genetic male (ZZ) gonads, which suggests that a robust genetic program is realized during female sex determination in *Xenopus*. However, a contrasting expression pattern was observed at later stages (NF56 and NF62), when the ZW gonads expressed less sex-specific genes than ZZ gonads, i.e., more genes may be involved in further development of the male gonads (ZZ). We identified sexual dimorphism in the expression of several functional groups of genes, including signaling factors, proteases, protease inhibitors, transcription factors, extracellular matrix components, extracellular matrix enzymes, cell adhesion molecules, and epithelium-specific intermediate filaments. In addition, our analysis detected a sexually dimorphic expression of many uncharacterized genes of unknown function, which should be studied further to reveal their identity and if/how they regulate gonad development, sex determination, and sexual differentiation. Comparison between genes sex-specifically expressed in developing gonads of *Xenopus* and available transcriptome data from zebrafish, two reptile species, chicken, and mouse revealed significant differences in the genetic control of sex determination and gonad development. This shows that the genetic control of gonad development is evolutionarily malleable.

**Electronic supplementary material:**

The online version of this article (10.1007/s00427-019-00630-y) contains supplementary material, which is available to authorized users.

## Introduction

*Xenopus laevis* is a good model to study molecular mechanisms of gonad development because the structural changes in developing gonads and the master gene determining sex, the W-linked DM-domain gene (*dm-w*), are well known. The *dm-w* is located on W chromosome and thus is present only in the genetic females (ZW) (Yoshimoto et al. 2008). At the earliest stage of gonad development, the gonads are undifferentiated and bipotential. The expression of *dm-w* triggers ovary development, while its absence promotes testis development. It is believed that the DM-W protein blocks the DMRT1 (doublesex and mab-3-related transcription factor 1) involved in male sex determination (Yoshimoto et al. 2010). In addition to the *dm-w*, many other genes, which act independently or downstream of *dm-w*, are involved in the development of bipotential gonads into the ovaries or the testes (Piprek et al. 2016). However, the expression and role of many genes involved in gonadal development is still vague. At the initial stage of gonadogenesis (NF50, Nieuwkoop-Faber stage 50), the gonads consist of the gonadal cortex and the medulla. The gonadal cortex contains coelomic epithelium and the germ cells, which adhere to the interior face of the epithelium. The medulla is sterile and contains medullar cells only (Piprek et al. 2016, 2017). At this stage, the sex-determining genes (*dm-w* and *dmrt1*) are expressed in the somatic cells of the gonads. In the absence of *dm-w*, i.e., in the differentiating testis (ZZ), around stage NF53, the cortex and medulla fuse. Subsequently, around stage NF56, the germ cells become enclosed by the somatic cells, which results in the formation of testis cords (Piprek et al. 2017). The typical structure of the testis, i.e., fully differentiated testis cords separated by the interstitium, is established at stage NF62. In contrast, in differentiating ovaries, which express *dm-w*, the germ cells remain in the cortical position, and at stage NF56, the ovarian cavity forms inside the gonad. Around NF62, the ovaries are fully differentiated, with the oocytes located in the cortex (Piprek et al. 2017; Yoshimoto et al. 2008). This divergent development of the female and male gonads has to be controlled by differential gene expression. A global analysis of *Xenopus* gonad transcriptome, which we performed in this study, is the step in obtaining a broad database of gene expression pattern in developing male and female *Xenopus* gonads.

Among vertebrates, the transcriptome of developing gonads has been studied in the mouse (Beverdam and Koopman 2006; Chen et al. 2012; Gong et al. 2013; Jameson et al. 2012; Nef et al. 2005; Small et al. 2005), chicken (Ayers et al. 2015; Scheider et al. 2014), slider *Trachemys scripta* (Czerwinski et al. 2016), *Alligator mississippiensis* (Yatsu et al. 2016), and in several teleost fish species (Bar et al. 2016; Lin et al. 2017; Sreenivasan et al. 2008; Sun et al. 2018; Xu et al. 2016). These studies provided valuable insights into the genes involved in gonad development and identified new sex-determining gene candidates.

**Fig. 1:**
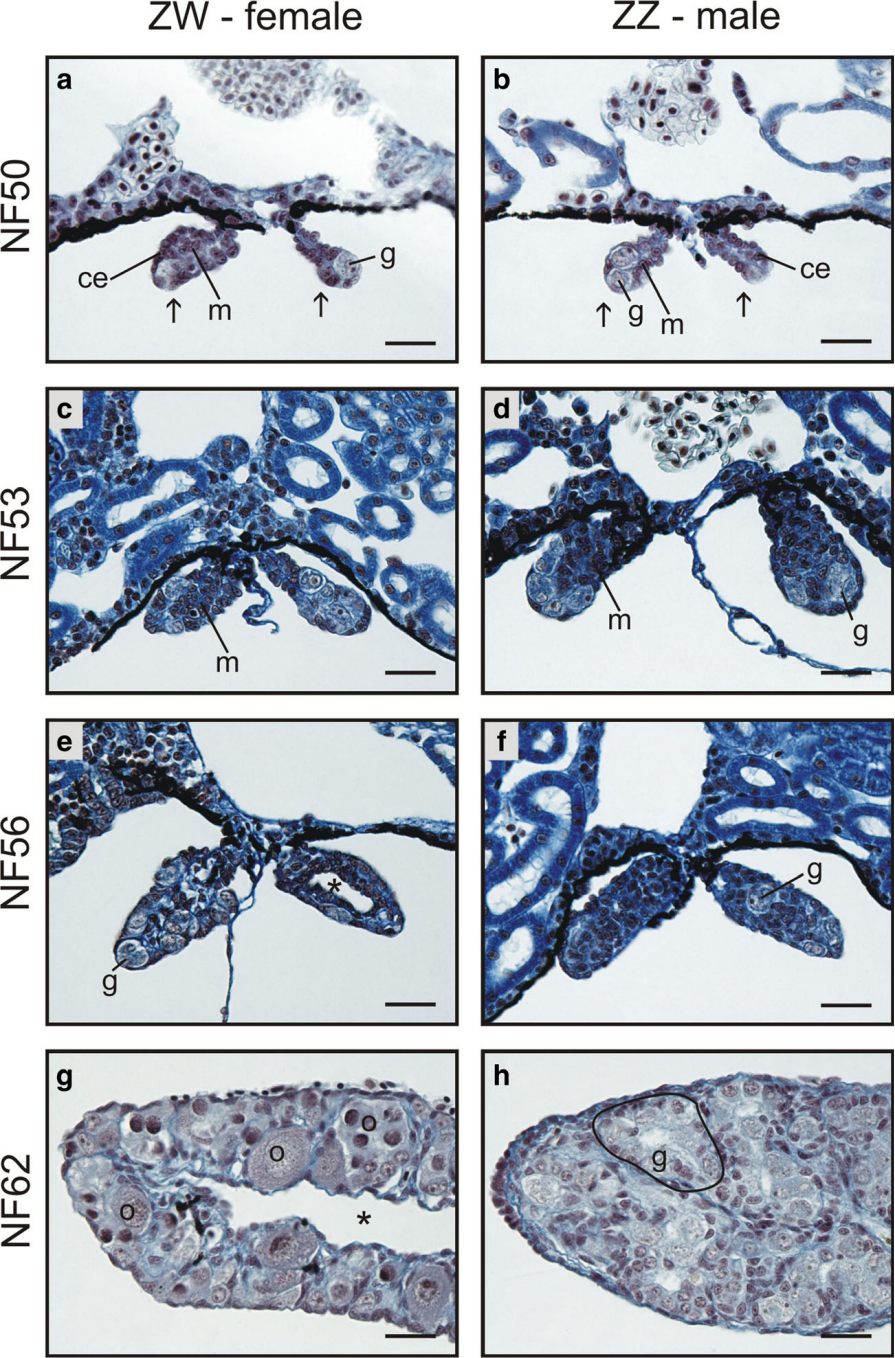
Structural changes in developing gonads. **a**, **b** At stage NF50, there is no difference in the gonad structure between genetic sexes (ZW and ZZ). Such undifferentiated gonads (arrows) are composed of the somatic cells of coelomic epithelium (ce) covering the gonad, and germ cells (g) located inside; the germ cells are attached to the coelomic epithelium. The somatic cells gather in the gonad center forming gonadal medulla (m). At stage NF53, the first sexual differences appear in the gonad structure; in the differentiating ovaries (**c**, ZW), the germ cells remain in the peripheral position forming the ovarian cortex, whereas the centrally located medulla remains sterile. In the ZZ (male) gonads at the onset of sexual differentiation (**d**, the onset of the testis differentiation), the germ cells (g) detach from the coelomic epithelium and move towards the gonad center (medulla, m). At stage NF56, the differentiating ovaries (**e**) becomes compartmentalized into cortex and medulla; all germ cells (g) are located in the cortex and are attached to the coelomic epithelium; an ovarian cavity forms in the medulla (asterisk). In the differentiating testes (**f**), the germ cells (g) are dispersed and the cortex and medulla are absent. At stage NF62, the ovaries (**g**) contain large ovarian cavity (asterisk); the ovarian cortex contains meiotic cells (o). In the testes (**h**), the germ cells (g) are located within the testis cords (encircled). Scale bar, 25 μm

Among anurans, a transcriptome analysis was performed only in *Silurana* (*Xenopus*) *tropicalis* and only on already sexually differentiated gonads (from stage NF58) (Haselman et al. 2015). Thus, the genes expressed before and during the sexual differentiation of amphibian gonads are still unknown. The aim of our study was to examine the transcriptome of developing *Xenopus* gonads from the earliest stage of gonad development. We studied the gene expression pattern in four different stages of gonad development: the undifferentiated gonad during the period of sex determination (NF50), gonads at the onset of sexual differentiation (NF53), the differentiating gonads (NF56), and during the developmental progression of differentiated gonads (NF62) (Fig. 1).

## Results and discussion

### Sex-specific changes in the level of gene expression

**Fig. 2:**
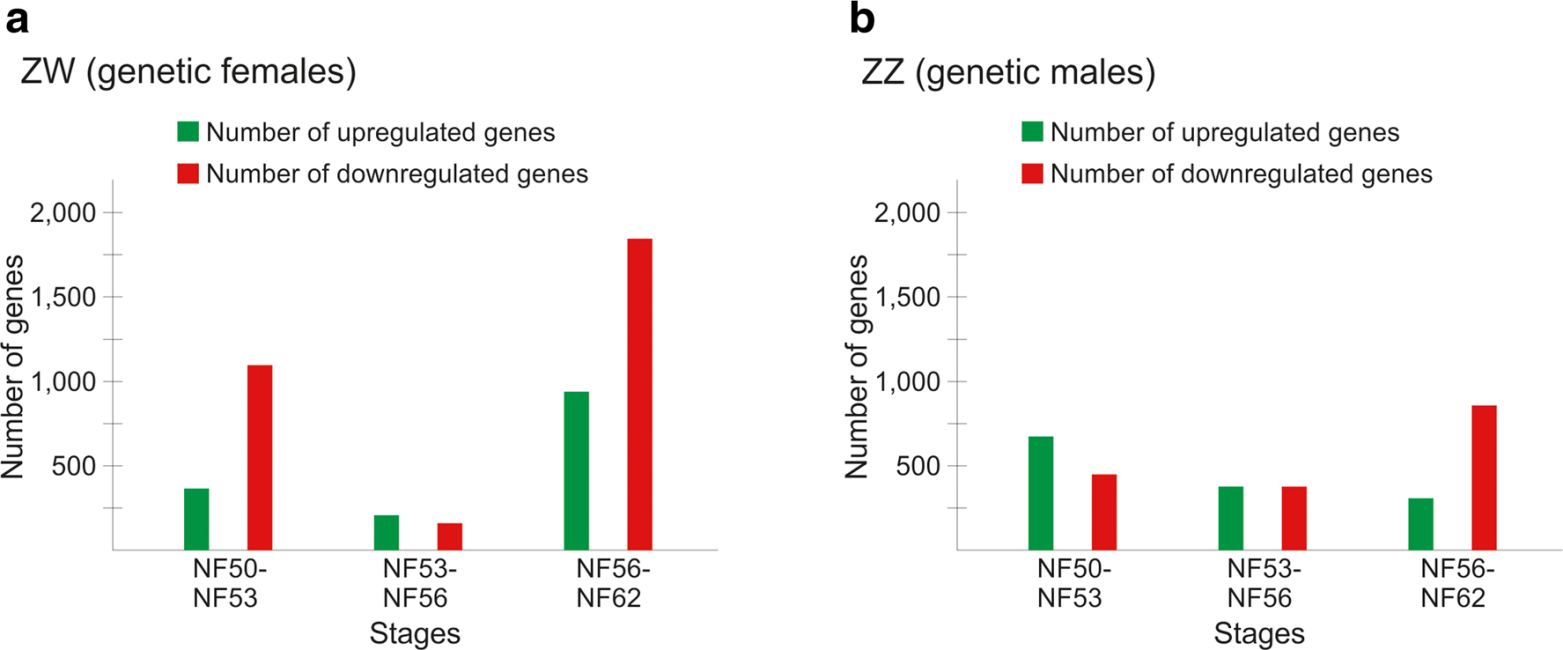
Diagram of changes in the number of genes upregulated and downregulated (≥ 2-fold change) between different stages in ZW gonads (**a**) and ZZ gonads (**b**)

**Fig. 3:**
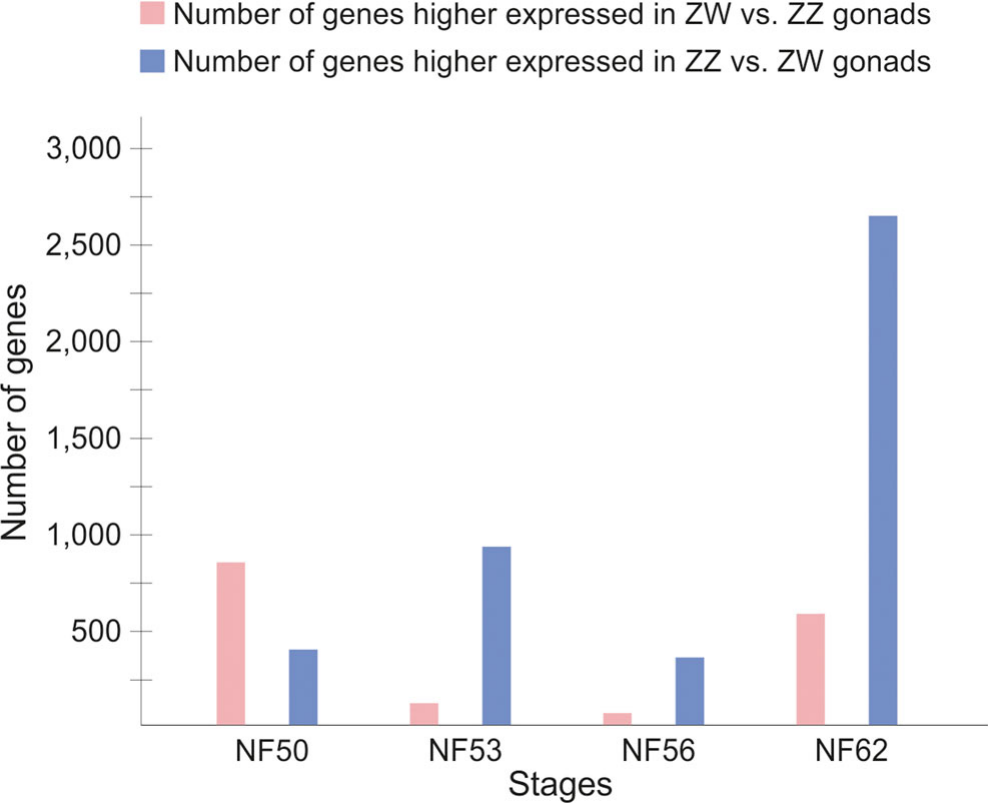
Diagram of changes in the number of genes with higher expression in ZW or ZZ gonads (≥ 2-fold change)

In developing *Xenopus laevis* gonads (stages NF50, NF53, NF56, and NF62 combined), we detected the expression of 63,084 transcripts in total. We found that while the expression level of the majority of genes was similar between stages and between male and female gonads, a subpopulation of genes showed distinct changes in the expression level between stages and sexes, which suggested that they may play a role in sex determination and/or sexual differentiation (Figs. 2A, B and 3, Tables 1 and 2).

**Table 1:** Number of genes with up- and downregulated (≥ 2-fold change) expression in ZW and ZZ gonads

Compared stages	ZW (females)		ZZ (males)	
	Upregulated	Downregulated	Upregulated	Downregulated
NF53 vs. NF50	376	1078	659	436
NF56 vs. NF53	143	128	340	340
NF62 vs. NF56	918	1834	334	831

**Table 2:** Number of genes with up- and downregulated (≥ 2-fold change) expression in ZW versus ZZ gonads

ZW vs. ZZ compared at stages	Upregulated in ZW	Downregulated in ZW
NF50	820	372
NF53	193	890
NF56	75	346
NF62	594	2630

Analysis of gene expression level in the gonads showed that in the genetic females (ZW), the gonads at the onset of sexual differentiation (NF53) had 376 genes with upregulated expression and 1078 genes with downregulated expression in comparison to the undifferentiated gonad during sex determination period (NF50) (Fig. 2, Table 1). In the differentiating ovaries (NF56), only 143 genes were upregulated and 128 genes were downregulated in comparison to NF53 (Table 1). In differentiated ovaries (NF62), there were 918 genes with upregulated expression and 1834 genes with downregulated expression in comparison to NF56 (Table 1).

The genetic male (ZZ) gonads at the onset of sexual differentiation (NF53) had 659 genes with upregulated expression and 436 genes with downregulated expression in comparison to NF50 stage (Fig. 2, Table 1). In differentiating testes (NF56), 340 genes were up-, and 340 downregulated in comparison to NF53 stage. The differentiated testes at stage NF62 had 334 genes with upregulated expression and 831 genes with downregulated expression in comparison to NF56 stage.

Altogether, these data indicate that in both sexes, the transcriptional regulation is more robust during early gonadal development, i.e., at the onset of sexual differentiation of the gonad (NF50-NF53) and in the already differentiated gonads NF56-NF62 than in the differentiating gonads (NF53-NF56).

The comparison of gene expression level in between ZW and ZZ gonads showed significant differences between the sexes and revealed sexually dimorphic pattern of gene expression. At the initial phase of gonad development, i.e., in the undifferentiated gonads during sex determination (NF50), there were 1192 genes (i.e., 3.4%) with sexually dimorphic expression (≥ 2-fold change). Eight hundred twenty genes showed higher expression in ZW (genetic females), and only 372 showed higher expression in ZZ (genetic males) gonads (Fig. 3, Table 2). This indicates that female sex determination in *Xenopus* involves a robust transcriptional regulation. In contrast, in mice, during the sex determination period (between embryonic day E10.5 and E12.5), a higher number of genes were upregulated in the XY (genetic males) than in the XX (genetic females) gonads (Nef et al. 2005), which suggested that programs of sex determination may be diverse among vertebrates.

Our analysis showed that at NF53, i.e., at the beginning of sexual differentiation of *Xenopus* gonads, 1083 genes (i.e., 3%) showed sexually dimorphic expression (≥ 2-fold change), which was slightly lower number than at NF50 (during sex determination). One hundred ninety-three genes showed higher expression in ZW gonads, and 890 in ZZ gonads (Fig. 3, Table 2). Thus, at the onset of sexual differentiation, more genes were specifically expressed in ZZ (male) gonads than in ZW (female) gonads in *Xenopus*, which was opposite to the mouse, where more genes were specifically expressed in XX (female) than XY (male) gonads at the beginning of sexual differentiation (E13.5) (Nef et al. 2005). This again indicates differences in the molecular programs of gonad development among vertebrates.

At NF56, i.e., in the differentiating gonads, only 421 genes (i.e., 1.2%) showed sexually dimorphic expression (≥ 2-fold change). This stage showed the lowest percentage of genes with sexually dimorphic expression among all stages. Seventy-five genes had higher expression in ZW, and 346 in ZZ gonads (Fig. 3, Table 2). Thus, more genes were highly expressed in ZZ gonads (differentiating testes) than in ZW (differentiating ovaries). We previously showed that the testis differentiation in *Xenopus* is a complex process during which the basement membranes between gonadal cortex and medulla disintegrate, the cortex and medulla fuse, and the germ cells and somatic cells gather to form the testis cords (Piprek et al. 2017). This sequence of profound structural changes certainly requires an involvement of a number of different genes, which is reflected in the high number of genes expressed in ZZ gonads at this stage.

At stage NF62, the sexual dimorphism of gene expression is the most pronounced. At this stage, 3224 genes (i.e., 5%) showed sexually dimorphic expression (≥ 2-fold change). However, only 594 genes showed higher expression in ZW (ovaries), and as many as 2630 in ZZ (testes) gonads. This is the stage when the gonads of both sexes are already differentiated and fully prepared to perform their sex-specific functions, and therefore the sexual dimorphism is evident not only at structural but also at molecular level.

### The expression of genes during different stages of ovary development

We found that in ZW gonads at stage NF53, in comparison to stage NF50, 376 genes had upregulated expression. The list of genes is presented in Suppl. Table 1, and chosen genes are presented in Table 3. Functional analysis grouped these genes in several distinct categories shown in Table 4. Among the upregulated genes, monoacylglycerol O-acyltransferase 2 gene 1 (*mogat2.1*) is involved in synthesis of diacylglycerol (DAG) that acts as a messenger lipid in cell signaling (Toker 2005); retinol-binding protein 2 (*rbp2*) is involved in retinoic acid regulation; extracellular proteins: collagen 2 and collagen 9, cysteine protease cathepsin K, epithelium-specific intermediate filaments: keratin 14 and keratin 19, estrogen receptor 1 (*esr1*), and synuclein gamma. At this early stage, the germ and somatic cells proliferate, and somatic cells start gathering in the gonad center forming medulla (Fig. 1A, C). Collagens accumulate between the gonad cortex and medulla (Piprek et al. 2017). Importantly, around stage NF50, a sex determination period takes place and gene expression analysis suggest that DAG, retinol, and estradiol may be involved in *Xenopus* sex determination.

**Table 3:** Chosen genes up- and downregulated in ZW (genetic females) gonads at NF53 in relation to NF50 stage

Probe name	Gene symbol	Gene name	Log FC
Genes upregulated (higher expression at NF53 than at NF50)			
A_10_P009259	*mogat2.1*	Monoacylglycerol O-acyltransferase 2.1	6.53907
A_10_P079665	*rbp2*	Retinol-binding protein 2	5.67257
A_10_P002950	*col9a1*	Collagen, type IX, alpha 1	4.86313
A_10_P005551	*srpx2*	Sushi repeat–containing protein, X2	4.74263
A_10_P000515	*bcan*	Brevican	4.008
A_10_P136703	*krt14*	Keratin 14	3.56258
A_10_P007276	*aldh3b1*	Aldehyde dehydrogenase 3 B1	3.5464
A_10_P143593	*ctsh*	Cathepsin H	3.39144
A_10_P004976	*matn4*	Matrilin 4	3.3843
A_10_P027124	*col2a1b*	Collagen, type II, alpha 1	3.12339
A_10_P002931	*matn2*	Matrilin 2	3.10895
A_10_P041821	*sncg-b*	Synuclein, gamma b	2.79753
A_10_P032181	*sncg-a*	Synuclein, gamma a	2.7756
A_10_P046256	*ctsk*	Cathepsin K	2.75751
A_10_P165493	*krt19*	Keratin 19	2.48345
A_10_P006607	*col9a3*	Collagen, type IX, alpha 3	2.41836
A_10_P033056	*esr1-a*	Estrogen receptor 1	2.36005
A_10_P224323	*racgap1*	Rac GTPase activating protein 1	2.29739
A_10_P036156	*dcn*	Decorin	2.25563
A_10_P065984	*itga11*	Integrin, alpha 11	2.17377
Genes downregulated (higher expression at NF50 than at NF53)			
A_10_P174228	*chrd*	Chordin	11.53231
A_10_P030946	*rbp4*	Retinol-binding protein 4	6.862097
A_10_P056207	*vtn*	Vitronectin	6.558013
A_10_P075910	*serpini2*	Serpin peptidase inhibitor, clade I .2	5.739304
A_10_P008816	*serpina3*	Serpin peptidase inhibitor, clade A .3	4.968027
A_10_P065884	*wnt10b*	Wingless-type MMTV integration site 10B	4.090623
A_10_P002182	*serpinc1*	Serpin peptidase inhibitor, clade C .1	3.044408
A_10_P009298	*igf3*	Insulin-like growth factor 3	3.030882
A_10_P043816	*dmrt2*	Doublesex and mab-3 related transcription factor 2	2.872563
A_10_P178123	*mafb*	v-maf avian musculoaponeurotic fibrosarcoma oncogene homolog B	2.66641

**Table 4:** Number of genes assigned to functional groups up- and downregulated in ZW (genetic female) gonads

Functional gene groups	ZW (genetic females)					
	NF53 vs. NF50		NF56 vs. NF53		NF62 vs. NF56	
	Up	Down	Up	Down	Up	Down
Signaling factors	20	61	7	8	–	103
Calcium-binding proteins	6	–	3	–	–	–
Iron-binding proteins	4	–	–	–	–	–
Monooxygenases	4	–	–	–	–	11
Oxidoreductases	5	11	–	–	–	22
Sushi domain–containing proteins	2	–	–	–	–	–
Metalloproteinases	3	–	–	–	–	8
Intermediate filaments	3	–	–	–	–	–
EGF-like domain–containing proteins	3	–	–	–	–	–
ECM-receptor interaction pathway	3	–	–	–	–	–
Progesterone-mediated oocyte maturation pathway	4	–	–	–	11	–
Proteases	–	12	–	–	–	18
Hydrolases	–	27	–	–	–	33
Disulfide bond–containing proteins	–	–	5	–	–	45
Extracellular matrix components	–	–	–	5	–	–
Markers of epithelial differentiation	–	–	–	2	–	–
Meiosis regulation factors	–	–	–	–	8	–
RNA-binding proteins	–	–	–	–	15	–
Phosphoproteins	–	–	–	–	16	–
Proteins involved in development	–	–	–	–	22	–
Proteins involved in oogenesis	–	–	–	–	3	–
Cytoplasmic proteins	–	–	–	–	35	–
Cytoskeletal proteins	–	–	–	–	12	–
Proteins involved in differentiation	–	–	–	–	9	–
Nuclear proteins	–	–	–	–	45	–
Transcriptional repressors	–	–	–	–	8	–
DNA-binding proteins	–	–	–	–	3	–
Oocyte meiosis	–	–	–	–	10	–
p53 signaling	–	–	–	–	6	–
Basal transcription factors	–	–	–	–	4	–
Proteins involved in DNA Replication	–	–	–	–	4	–
Proteins involved in the formation of dorso-ventral axis	–	–	–	–	3	–
Secreted proteins	–	–	–	–	–	23
Transport proteins	–	–	–	–	–	36

We also found that in ZW gonads at stage NF53, there were 1078 genes with a downregulated expression in comparison to stage NF50. All these genes are listed in Suppl. Table 2, and chosen genes are presented in Table 3. Functional analysis grouped these genes in four categories shown in Table 4. Among these downregulated genes, there were signaling protein chordin (*chrd*), retinol-binding protein (*rbp4*), several protease inhibitors serpins, signaling proteins *wnt10b* and *igf3* (insulin-like growth factor 3), transcription factors *dmrt2*, and *mafb* (Table 3).

In developing ZW gonad at stage NF56, in comparison to stage NF53, there were 143 genes with upregulated expression (Suppl. Table 3, and chosen genes are presented in Table 5). Functional analysis grouped these genes in three categories shown in Table 4. One of important genes upregulated in this period is a neurotrophin receptor a-1 (*p75NTRa*) (Table 2); its role in gonad development has never been studied; however, its upregulation suggests that neurotrophins (ligands of this receptor) can play a role in ovarian differentiation. We also found that in ZW gonad at stage NF56, in comparison to stage NF53, there were 128 genes with downregulated expression (Suppl. Table 4, and chosen genes are presented in Table 5). Functional analysis grouped these genes in several categories shown in Table 4. At NF56 stage, more genes responsible for reorganization of extracellular matrix and epithelial differentiation in ZW gonads are expressed than at stage NF53. Between stages NF53 and NF56, the medulla cells disperse, which results in the formation of the cavity in the ovary center (Fig. 1E). The mechanism of this event is not known and would be interesting to study how the neurotrophins, extracellular matrix, and epithelial differentiation are involved in this process.

**Table 5:** Chosen genes up- and downregulated in ZW (genetic females) gonads at NF56 in relation to NF53 stage

Probe name	Gene symbol	Gene name	Log FC
Genes upregulated (higher expression at NF56 than at NF53)			
A_10_P259017	*sag*	Arrestin	3.6903
A_10_P000364	*p75NTRa*	p75 neurotrophin receptor a-1	3.24871
Genes downregulated (higher expression at NF53 than at NF56)			
A_10_P000515	*bcan*	Brevican	3.073221
A_10_P136703	*krt14*	Keratin 14	2.897822
A_10_P004976	*matn4*	Matrilin 4	2.768856
A_10_P002950	*col9a1*	Collagen, type IX, alpha 1	2.713584
A_10_P140568	*krt5.6*	Keratin 5, gene 6	2.618006
A_10_P006607	*col9a3*	Collagen, type IX, alpha 3	2.601338
A_10_P084685	*krt14*	Keratin 14	2.530431
A_10_P038721	*col2a1b*	Collagen, type II, alpha 1	2.509659
A_10_P032181	*sncg-a*	Synuclein, gamma a	2.497221

In developing ZW gonad at stage NF62, in comparison to stage NF56, there were 918 genes with upregulated expression (Suppl. Table 5, and chosen genes are presented in Table 6). Functional analysis grouped these genes in the many categories (Table 4). Among known genes upregulated in the ovaries at stage NF62 are genes involved in meiosis and oocyte development, such as poly(A)-binding protein, oocyte-specific *pou5f3.3*, zygote arrest 1, zona pellucid proteins (*zp2*, *zpd*, *zpy1*), *sycp3* (synaptonemal complex protein 3),and *lhx8* (LIM homeobox 8). This reflects the onset of meiosis at stage NF62 and appearance of first oocytes (Fig. 1G). Also, more genes involved in the regulation of development, such as genes encoding the following: vegt protein, growth differentiation factor (*gdf1*), *foxh1*, *foxr1*, *wnt11b*, *ddx25*, and the survivin which prevents apoptosis, were upregulated at stage NF62 than at stage NF56.

**Table 6:** Chosen genes up- and downregulated in ZW (genetic females) gonads at NF62 in relation to NF56 stage

Probe name	Gene symbol	Gene name	Log FC
Genes upregulated (higher expression at NF62 than at NF56)			
A_10_P000661	*spdyc-b*	Speedy/RINGO cell cycle regulator C	5.91483
A_10_P041271	*pabpn1l-a*	Poly(A) binding protein, nuclear 1-like	5.78779
A_10_P078660	*rnf138*	Ring finger protein 138	5.43076
A_10_P004355	*pou5f3.3*	POU class 5 homeobox 3, gene 3	4.82381
A_10_P002029	*zar1*	Zygote arrest 1	4.7962
A_10_P038461	LOC398389	Survivin	4.75826
A_10_P027361	*vegt-a*	vegt protein	4.68137
A_10_P007276	*aldh3b1*	Aldehyde dehydrogenase 3 family, B1	4.65557
A_10_P032511	*cldn6.1*	Claudin 6, gene 1	4.50308
A_10_P162298	*zp2*	Zona pellucida glycoprotein 2	4.43055
A_10_P009533	*gdf1*	Growth differentiation factor 1	4.40831
A_10_P002027	*velo1*	velo1 protein	4.36483
A_10_P027280	*zpd*	Zona pellucida protein D	4.2713
A_10_P205908	*foxh1*	Forkhead box H1	4.2256
A_10_P031016	*foxr1*	Forkhead box R1	4.10517
A_10_P008731	*wnt11b*	Wingless-type MMTV integration site family, member 11B	4.0833
A_10_P033516	*zpy1*	Zona pellucida protein Y1	4.00754
A_10_P117061	*ddx25*	DEAD box helicase 25	3.89223
A_10_P040816	*sycp3*	Synaptonemal complex protein 3	3.70889
A_10_P071715	*lhx8*	LIM homeobox 8	3.56271
A_10_P056732	*dppa2*	Developmental pluripotency-assoc 2	3.51303
A_10_P027350	*adam21*	ADAM metallopeptidase domain 21	2.89064
Genes downregulated (higher expression at NF56 than at NF62)			
A_10_P047196	LOC100037217	Uncharacterized LOC100037217	6.582348
A_10_P180718	*hrg*	Histidine-rich glycoprotein	6.249551
A_10_P004053	*rbp4*	Retinol-binding protein 4	5.794043
A_10_P034336	*serpina1*	Serpin peptidase inhibitor, A1	5.541168
A_10_P006319	*sag*	Arrestin	5.153979
A_10_P075910	*serpini2*	Serpin peptidase inhibitor, I2	4.285183
A_10_P030976	LOC398504	Villin-1-like	3.897723
A_10_P068493	*fetub*	Fetuin B	3.871496
A_10_P110124	*krt12*	Keratin 12	3.5294
A_10_P006916	*emx1.2*	Empty spiracles homeobox 1, gene 2	3.507484
A_10_P002103	*mmp7*	Matrix metallopeptidase 7	3.50358
A_10_P153143	*igf3*	Insulin-like growth factor 3	3.452683
A_10_P003788	*igfbp1-a*	Insulin-like growth factor–binding 1	3.06992
A_10_P005507	*ctsl*	Cathepsin L	2.882569
A_10_P137683	*gata2*	GATA binding protein 2	2.5687
A_10_P053899	*cdh26*	Cadherin 26	2.529154
A_10_P126889	*rdh16*	Retinol dehydrogenase 16 (all-trans)	2.355785
A_10_P174228	*chrd*	Chordin	2.347432
A_10_P007857	*timp2*	TIMP metallopeptidase inhibitor 2	2.066979

In developing ZW gonad at stage NF62, in comparison to stage NF56, there were 1834 genes with downregulated expression (Suppl. Table 6, and chosen genes are presented in Table 6). Functional analysis grouped these genes into several categories (Table 4). Also, many (24) pathways were downregulated, including metabolic pathways, steroid hormone biosynthesis, retinol metabolism, PPAR signaling pathway, and adipocytokine signaling pathway (Table 4). Among known genes downregulated in the ovaries at stage NF62 are the following genes: retinol-binding protein 4 (*rbp4*), *rdh16* (retinol dehydrogenase 16), several serpins, *emx1.2* (empty spiracles homeobox 1), *igf3* (insulin-like growth factor 3), *igfbp1-a* (insulin-like growth factor–binding protein 1), *gata2* (GATA binding protein 2), and chordin. This indicates that retinol pathway and insulin-like growth factor pathway are downregulated at a later stage of ovarian development (NF62), and that these two pathways may be important for earlier stages of ovarian development. The PPAR signaling pathway and adipocytokine signaling pathway are involved in fat tissue differentiation (Ogunyemi et al. 2013) and are probably important for the development of corpora adiposa (fat tissue) at the anterior edges of the developing gonads at stages before NF62. Thus, after the fat tissue had been formed, these pathways are downregulated at stage NF62.

Another interesting gene expressed at the onset of gonadogenesis (NF50), showing upregulation at NF53 and downregulated at NF62 is chordin (*chrd*). Several studies showed that this gene is crucial for early organogenesis (dorsalization, gastrulation, and head development (Pappano et al. 1998; Bachiller et al. 2000), but its role in gonad development is unknown. Overall, our gene expression analysis showed that the later development of the ovary (NF62) is a very transcriptionally active period (many genes become upregulated and downregulated between NF56 and NF62), which may be related to the initialization of meiosis and oocyte formation during this developmental period.

### The expression of genes during different stages of testis development

Our analysis showed that in the genetic male (ZZ) gonads at stage NF53, i.e., at the beginning of sexual differentiation, there were 659 genes with upregulated expression in comparison to the stage NF50 gonad (Suppl. Table 7, and chosen genes are presented in Table 7). Functional analysis grouped these genes into several categories (Table 8). There were the following genes with known function: *igf3* (insulin-like growth factor 3), *rbp4* (retinol-binding protein 4), *vtn* (vitronectin), several serpins, *esr2* (estrogen receptor 2), several components of extracellular matrix (collagen 9, matrilin 2), and extracellular matrix (*timp3*) enzymes. A role of these genes in the early phase of ZZ gonad development is not known, and it would be interesting to study if retinol and/or *igf3* are involved in male sex determination in *Xenopus*. Upregulation of PPAR and adipocytokine signaling pathways, characteristic for fat tissue, possibly reflects the onset of the development of the fat bodies at the anterior edge of the gonad.

**Table 7:** Chosen genes up- and downregulated in ZZ (genetic males) gonads at NF53 in relation to NF50 stage

Probe name	Gene symbol	Gene name	Log FC
Genes upregulated (higher expression at NF53 than at NF50)			
A_10_P030946	*rbp4*	Retinol-binding protein 4	4.97523
A_10_P056207	*vtn*	Vitronectin	4.51992
A_10_P075910	*serpini2*	Serpin peptidase inhibitor, clade I. 2	4.4381
A_10_P041856	*igf3*	Insulin-like growth factor 3	4.34284
A_10_P003882	*timp3*	TIMP metallopeptidase inhibitor 3	2.37097
A_10_P007964	*serpinf2*	Serpin peptidase inhibitor, F2	2.32024
A_10_P030126	*esr2*	Estrogen receptor 2 (ER beta)	2.28938
A_10_P058537	*col9a1-b*	Collagen, type IX, alpha 1	2.24147
A_10_P048579	*ocln-b*	Occludin	2.22878
A_10_P002931	*matn2*	Matrilin 2	2.06945
Genes downregulated (higher expression at NF50 than at NF53)			
A_10_P017957	*ocm*	Oncomodulin	6.204741
A_10_P140568	*krt5.6*	Keratin 5, gene 6	4.866387
A_10_P138508	*krt15*	Keratin 15	4.154655
A_10_P126949	*mmp1*	Matrix metallopeptidase 1	2.745253
A_10_P008082	*fgfbp1*	Fibroblast growth factor–binding 1	2.66819
A_10_P203798	*lum*	Lumican	2.460273
A_10_P222743	*isyna1-b*	Inositol-3-phosphate synthase 1	2.399986
A_10_P002391	*capn8-a*	Calpain 8	2.388139
A_10_P040276	*wnt7b*	Wingless-type MMTV integration site family, member 7B	2.038541

**Table 8:** Number of genes assigned to functional groups up- and downregulated in ZZ (genetic male) gonads

Functional gene groups	ZZ (genetic males)					
	NF53 vs NF50		NF56 vs NF53		NF62 vs NF56	
	Up	Down	Up	Down	Up	Down
Signaling factors	48	–	13	43	17	–
Calcium-binding proteins	–	–	5	–	–	–
Metal-binding proteins	30	–	–	21	–	–
Monooxygenases	–	–	–	–	3	8
Oxidoreductases	9	–	–	8	5	14
Metalloproteinases	4	–	–	3	–	–
EGF-like domain–containing proteins	4	–	–	–	–	–
Proteases	13	–	–	14	9	–
Hydrolases	20	–	–	25	12	–
Disulfide bond–containing proteins	35	–	–	31	13	–
Secreted proteins	–	–	–	12	–	–
Transport proteins	–	3	–	–	–	–
Steroid hormone synthesis pathway	4	–	–	–	2	–
Insulin signaling pathway	4	–	–	7	–	–
PPAR signaling pathway	4	–	–	3	–	–
Adipocytokine signaling pathway	5	–	–	6	–	–
Mitochondrial proteins	–	7	–	–	–	–
Ion transport	–	5	–	–	–	–
Terpenoid backbone biosynthesis pathway	–	3	–	–	–	3
ER protein processing pathway	–	5	–	–	–	–
Receptors	–	–	6	–	–	–
Metabolic pathway	–	–	–	23	8	–
FoxO signaling pathway	–	–	–	7	–	45
Cell membrane proteins	–	–	–	–	–	48
Intercellular transport	–	–	–	–	–	21
Retinol metabolism	–	–	–	–	–	5

Our analysis also showed that in the genetic male (ZZ) gonads at stage NF53, there were 436 genes with downregulated expression (Suppl. Table 8, and chosen genes are presented in Table 7). Functional analysis grouped these genes in the categories shown in Table 8.

Comparison of gene expression level in the ZZ gonads between stage NF56 and NF53 showed that at stage NF56, there were 340 genes with upregulated expression (Suppl. Table 9, and chosen genes are presented in Table 9). Functional analysis grouped these genes in categories shown in Table 8. Some of these upregulated genes are *rbp2* (retinol-binding protein 2), receptor of prostaglandin E (*ptger3*), stromal cell-derived factor 2-like 1 (*sdf2l1*), and neurotrophin receptor (*p75NTRa*). Further, studies are necessary to establish what is the exact role of the prostaglandin E, retinol, and neurotrophins in testis differentiation. Importantly, around NF53-NF56, the cortex and medulla fuse in differentiating testes, and the germ cells lose their connection with the superficial coelomic epithelium and disperse in the whole testis (Fig. 1F). There were also 340 genes downregulated at stage NF56 ZZ gonad in comparison to stage NF53 gonad (Suppl. Table 10, and chosen genes are presented in Table 9). Functional analysis grouped these genes into several categories (Table 8).

**Table 9:** Chosen genes up- and downregulated in ZZ (genetic males) gonads at NF56 in relation to NF53 stage

Probe name	Gene symbol	Gene name	Log FC
Genes upregulated (higher expression at NF56 than at NF53)			
A_10_P079665	*rbp2*	Retinol-binding protein 2, cellular	6.44222
A_10_P043951	*ptger3*	Prostaglandin E receptor 3	2.75218
A_10_P036706	*sdf2l1*	Stromal cell-derived factor 2-like 1	2.32111
A_10_P000364	*p75NTRa*	p75 neurotrophin receptor a-1	2.02373
Genes downregulated (higher expression at NF53 than at NF56)			
A_10_P036346	LOC100189571	Uncharacterized LOC100189571	8.899836
A_10_P102465	*rbp4*	Retinol-binding protein 4	7.963364
A_10_P056207	*vtn*	Vitronectin	7.657184
A_10_P027027	*ptx*	Pentraxin	7.367071
A_10_P041856	*igf3*	Insulin-like growth factor 3	3.440657
A_10_P002182	*serpinc1*	Serpin peptidase inhibitor C1	2.826371
A_10_P094993	*krt12*	Keratin 12	2.032246

Comparison of gene expression level in the ZZ gonads between stages NF62 and NF56 showed that at stage NF62 gonad, there were 334 genes with the upregulated expression (Suppl. Table 11, and chosen genes are presented in Table 10). Functional analysis grouped these genes into several categories (Table 8). Around stage NF56-NF62, cells group into the testis cords (Fig. 1H). Genes involved in this process are not known, and presumably, the genes upregulated at this stage may be responsible for the formation of testis cords. There were also 831 genes downregulated in ZZ gonad at stage NF62 in comparison to stage NF56 (Suppl. Table 12, and chosen genes are presented in Table 10, and the gene categories, which were analyzed functionally are shown in Table 8).

**Table 10:** Chosen genes up- and downregulated in ZZ (genetic males) gonads at NF62 in relation to NF56 stage

Probe name	Gene symbol	Gene name	Log FC
Genes upregulated (higher expression at NF62 than at NF56)			
A_10_P049320	*prss1*	Protease, serine, 1	6.7897
A_10_P045961	*prss3*	Protease, serine, 3	6.64558
A_10_P259137	*tfip11*	Tuftelin-interacting protein 11	6.14112
A_10_P027545	*mmp11*	Matrix metallopeptidase 11	3.31378
A_10_P027246	*klf9-a*	Kruppel-like factor 9	2.7011
A_10_P203798	*lum*	Lumican	2.10476
Genes downregulated (higher expression at NF56 than at NF62)			
A_10_P032408	*ocm.2*	Oncomodulin	7.542298
A_10_P004053	*rbp4*	Retinol-binding protein 4	5.656075
A_10_P000084	*krt5.5*	Keratin 5, gene 5	4.410092
A_10_P003972	*mmp28-b*	Matrix metallopeptidase 28	3.191715
A_10_P044151	*fgfr4-b*	Fibroblast growth factor receptor 4	3.091348
A_10_P002657	*isyna1-a*	Inositol-3-phosphate synthase 1	3.030967
A_10_P094993	*krt12*	Keratin 12	2.838533

### Genes with sexual dimorphism of expression in ZW and ZZ gonads in different developmental stages

The master sex-determining gene in *Xenopus* the *dm-w* was discovered in 2008 (Yoshimoto et al. 2008), but the molecular machinery of sex determination is certainly very complex and contains many other genes. We previously published the expression profile of known genes involved in sex determination and sexual differentiation in the *Xenopus* gonads (Piprek et al. 2018). We showed that the *gata4*, *sox9*, *dmrt1*, a*mh*, *fgf9*, *ptgds*, *pdgf*, *fshr*, and *cyp17a1* had upregulated expression in testes, while *dm-w*, *fst*, *foxl2*, and *cyp19a1* had upregulated expression in the ovary (Piprek et al. 2018).

Here, we compared gene expression level between ZW and ZZ gonads at different stages of gonad development. These analyses showed that at stage NF50 (undifferentiated gonads during sex determination period), there were 820 genes with upregulated expression in ZW gonad (Suppl. Table 13, and chosen genes are shown in Table 12). Functional analysis grouped these genes into several categories (Table 11). Many genes upregulated in this period are uncharacterized. Among known genes upregulated in ZW gonad at stage NF50 is chordin (*chrd*). Chordin is a secreted protein responsible for several developmental processes such as dorsalization, head development, and gastrulation (Sasai et al. 1994; Pappano et al. 1998; Bachiller et al. 2000); our study indicates that it may play a crucial role in female sex determination (Table 12, Suppl. Table 13). Other genes upregulated in ZW gonad at NF50 are two protease inhibitors, serpin A3 and serpin I2, extracellular glycoprotein vitronectin, metalloproteinases *mmp7* and *adam27*, retinol-binding protein *rbp4*, signaling molecules *wnt10b*, *wnt11b*, and *igf3*, helicase *ddx25*, and transcription factors *foxa2* and *lhx8*. A role of these factors in sex determination in *Xenopus* is unknown and requires further study.

**Table 11:** Number of genes assigned to functional groups expressed at higher level in ZW and ZZ gonads

Functional gene groups	NF50		NF53		NF56		NF62	
	ZW	ZZ	ZW	ZZ	ZW	ZZ	ZW	ZZ
Signaling factors	64	18	–	50	18	–	–	73
Calcium-binding proteins	–	–	–	–	3	–	–	–
Metal-binding proteins	28	–	–	26	–	–	–	–
Metalloproteinases	7	–	–	–	–	–	–	–
Progesterone-mediated oocyte maturation pathway	–	–	–	–	–	–	8	–
Proteases	20	–	–	9	–	–	–	–
Hydrolases	28	–	–	21	–	–	–	25
Disulfide bond–containing proteins	42	–	–	34	10	6	–	52
Extracellular matrix components	–	3	–	–	–	–	–	–
Markers of epithelial differentiation	–	2	–	–	–	–	–	–
Meiosis regulation factors	–	–	–	–	–	–	4	–
Oocyte meiosis	–	–	–	–	–	–	7	–
RNA-binding proteins	–	–	–	–	–	–	11	–
Phosphoproteins	–	–	–	–	–	–	11	–
Proteins involved in development	–	–	–	–	–	–	18	19
Cytoplasmic proteins	–	–	–	–	–	–	30	–
Cytoskeletal proteins	–	–	–	–	–	–	10	–
Nuclear proteins	–	–	–	–	–	–	35	–
p53 signaling	–	–	–	–	–	–	6	–
Secreted proteins	15	7	–	14	6	–	–	19
Transport proteins	–	–	–	–	–	5	–	–
Metabolic pathway	14	–	–	33	–	–	–	–
Intermediate filaments	–	3	–	–	–	–	–	–
Mitochondrial proteins	–	5	–	–	–	–	–	–
Insulin signaling pathway	–	–	–	7	–	–	–	–
Steroid hormone synthesis	–	–	–	3	–	–	–	3
Adipocytokine signaling pathway	–	–	–	4	–	–	–	–
FoxO signaling pathway	–	–	–	8	–	–	–	–
Cell membrane proteins	–	–	–	–	–	5	–	63
Cell junction proteins	–	–	–	–	–	4	–	–
Ion channel proteins	–	–	–	–	–	4	–	–
Cell division proteins	–	–	–	–	–	–	10	–
Mitotic proteins	–	–	–	–	–	–	6	–
Wnt signaling pathway	–	–	–	–	–	–	–	5

**Table 12:** Chosen genes upregulated in ZW (genetic females) in relation to ZZ (genetic males) gonads at NF50 stage [higher gene expression level in ZW than in ZZ gonads]

Probe name	Gene symbol	Gene name	Log FC
A_10_P174228	*chrd*	Chordin	11.30213
A_10_P007346	MGC85508	MGC85508 protein	8.151194
A_10_P008816	*serpina3*	Serpin peptidase inhibitor, clade A3	6.774417
A_10_P075910	*serpini2*	Serpin peptidase inhibitor, clade I2	6.378762
A_10_P233398	*vtn*	Vitronectin	5.368433
A_10_P187778	*wnt11b*	Wingless-type MMTV integration site family, member 11B	5.00604
A_10_P004053	*rbp4*	Retinol-binding protein 4, plasma	4.876474
A_10_P065884	*wnt10b*	Wingless-type MMTV integration site family, member 10B	4.20504
A_10_P027350	*adam21*	ADAM metallopeptidase domain 21	3.851196
A_10_P009298	*igf3*	Insulin-like growth factor 3	3.848738
A_10_P202038	MGC69070	Matrix metalloproteinase 7	3.690095
A_10_P006376	*anxa13*	Annexin A13	3.483353
A_10_P003549	MGC69070	Matrix metalloproteinase 7	3.459862
A_10_P000388	*ddx25*	DEAD box helicase 25	3.239557
A_10_P082395	*foxa2*	Forkhead box A2	3.049031
A_10_P003648	*lhx8*	LIM homeobox 8	2.965778

There were 372 genes with higher expression level in the ZZ (genetic males) gonads at stage NF50 (Suppl. Table 14, and chosen genes are shown in Table 13, and the functional groups are shown in Table 11). Among these upregulated genes are known genes such as epithelium markers keratin 5, 12, and 14, coiled-coil domain containing 50 (*ccdc50*) that acts as an effector in EGF signaling and negative regulator of NF-kB factor (Tsukiyama et al. 2012), signaling molecules: wnt3a, wnt7b, growth differentiation factor 3 (*gdf3*), fibroblast growth factor–binding protein 1 (*fgfbp1*), proteases cathepsin K and H, extracellular matrix molecules lumican, collagen IX and I, and decorin. A role of these genes in male sex determination and early testis development remains unknown.

**Table 13:** Chosen genes downregulated in ZW in relation to ZZ gonads at NF50 stage [higher gene expression level in ZZ than in ZW gonads]

Probe name	Gene symbol	Gene name	Log FC
A_10_P136703	*krt14*	Keratin 14	7.50258
A_10_P183185	*ccdc50*	Coiled-coil domain containing 50	6.57626
A_10_P140568	*krt5.6*	Keratin 5, gene 6	5.84154
A_10_P003366	*lum*	Lumican	5.75697
A_10_P193923	*krt14*	Keratin 14	5.1494
A_10_P008082	*fgfbp1*	Fibroblast growth factor–binding protein 1	3.66899
A_10_P002950	*col9a1*	Collagen, type IX, alpha 1	3.07046
A_10_P046256	*ctsk*	Cathepsin K	2.60816
A_10_P036156	*dcn*	Decorin	2.60212
A_10_P244713	*col1a1*	Collagen, type I, alpha 1	2.56712
A_10_P040276	*wnt7b*	Wingless-type MMTV integration site family, member 7B	2.3506
A_10_P026995	*wnt3a*	Wingless-type MMTV integration site family, member 3A	2.25544
A_10_P094993	*krt12*	Keratin 12	2.23416
A_10_P000272	*gdf3*	Growth differentiation factor 3	2.07545
A_10_P046876	*ctsh*	Cathepsin H	2.01352

There are 193 genes with a higher expression in ZW (genetic females) gonad at stage NF53 (the onset of sexual differentiation of gonads) (Suppl. Table 15, and chosen genes are shown in Table 14). Functional analysis did not link these genes to any specific pathway. Among these upregulated genes, there are the following known genes: retinol-binding protein 2 (*rbp2*), protease calpain 8, synuclein gamma with unknown function, cell adhesion gene claudin 6, metalloproteinases mmp1 and adam21, and galectin-la involved in cell adhesion and signaling.

**Table 14:** Chosen genes upregulated in ZW in relation to ZZ gonads at NF53 stage [higher gene expression level in ZW than in ZZ gonads]

Probe name	Gene symbol	Gene name	Log FC
A_10_P079665	*rbp2*	Retinol-binding protein 2	5.229889
A_10_P032636	LOC100101274	Uncharacterized LOC100101274	3.804135
A_10_P062524	*lgalsia-a*	Galectin-Ia	2.939513
A_10_P008579	*krt5.2*	Keratin 5, gene 2	2.846329
A_10_P057292	*sncg-a*	Synuclein, gamma	2.52171
A_10_P002391	*capn8-a*	Calpain 8	2.404349
A_10_P032511	*cldn6.1*	Claudin 6, gene 1	2.207111
A_10_P027350	*adam21*	ADAM metallopeptidase domain 21	2.177794
A_10_P126949	*mmp1*	Matrix metallopeptidase 1	2.07391

There were 890 genes with higher expression in ZZ (genetic males) gonad at stage NF53 (the onset of sexual differentiation of gonads) (Suppl. Table 16, and chosen genes are shown in Table 15). Functional analysis grouped these genes into several categories (Table 11). The upregulated known genes are coiled-coil domain containing 50 (*ccdc50*), retinol-binding protein 4 (*rbp4*), signaling molecules *igf1* and *igf3*, estrogen receptor 2 (*esr2*), transcription factors, Kruppel-like factor 9 (*klf9*), Kruppel-like factor 15 (*klf15*), and *foxo1* (forkhead box O1), enzyme glycerophosphodiester phosphodiesterase 1 (*gde1*) responsible for synthesis of signaling molecule lysophosphatidic acid (LPA), cell adhesion proteins gap junction protein alpha 3 (*gja3*), occluding (*ocln*), and extracellular matrix component vitronectin (*vtn*).

**Table 15:** Chosen genes downregulated in ZW in relation to ZZ gonads at NF53 stage [higher gene expression level in ZZ than in ZW gonads]

Probe name	Gene symbol	Gene name	Log FC
A_10_P183185	*ccdc50*	Coiled-coil domain containing 50	7.79896
A_10_P009082	*gde1*	Glycerophosphodiester phosphodiesterase 1	6.52222
A_10_P030946	*rbp4*	Retinol-binding protein 4, plasma	5.8968
A_10_P233398	*vtn*	Vitronectin	5.85368
A_10_P009298	*igf3*	Insulin-like growth factor 3	3.50579
A_10_P002488	*gja3*	Gap junction protein, alpha 3, 46 kDa	3.2409
A_10_P001965	*klf15*	Kruppel-like factor 15	2.9746
A_10_P027246	*klf9-a*	Kruppel-like factor 9	2.74415
A_10_P030126	*esr2*	Estrogen receptor 2 (ER beta)	2.42444
A_10_P027093	*igf1*	Insulin-like growth factor 1	2.40431
A_10_P000763	*foxo1*	Forkhead box O1	2.14949
A_10_P048579	*ocln-b*	Occludin	2.12035

There were 75 genes with higher expression in ZW (genetic females) gonad at stage NF56 (Suppl. Table 17, and chosen genes are shown in Table 16, and the functional groups are shown in Table 11). Among known genes are retinoic binding protein 4 and vitronectin.

**Table 16:** Chosen genes upregulated in ZW versus ZZ gonads at NF56 stage [higher gene expression level in ZW than in ZZ gonads]

Probe name	Gene symbol	Gene name	Log FC
A_10_P036346	LOC100189571	Uncharacterized LOC100189571	5.456322
A_10_P056207	*vtn*	Vitronectin	4.026518
A_10_P030946	*rbp4*	Retinol-binding protein 4, plasma	3.555976

There were 346 genes with higher expression in ZZ (genetic males) gonad at stage NF56 (Suppl. Table 18, and chosen genes are shown in Table 17, and the functional groups are shown in Table 11). Among known genes are keratin 14 and 15, cell molecule gap junction protein, alpha (*gja3*), endophilin B2 (*sh3glb2*) and coiled-coil domain containing 50 (*ccdc50*).

**Table 17:** Chosen genes downregulated in ZW in relation to ZZ gonads at NF56 stage [higher gene expression level in ZZ than in ZW gonads]

Probe name	Gene symbol	Gene name	Log FC
A_10_P084685	*krt14*	Keratin 14	3.53568
A_10_P171263	*sh3glb2*	SH3-domain GRB2-like endophilin B2	3.50581
A_10_P183185	*ccdc50*	Coiled-coil domain containing 50	3.3565
A_10_P138508	*krt15*	Keratin 15	3.13432
A_10_P002488	*gja3*	Gap junction protein, alpha 3, 46 kDa	2.0898

There were 594 genes with higher expression in ZW (genetic females) gonad at stage NF62 (Suppl. Table 19, and chosen genes are shown in Table 18, and the functional groups are shown in Table 11). Many genes expressed at this stage such as zona pellucida glycoprotein 4 (*zp4*) and zona pellucida C glycoprotein (*xlzpc*) are involved in ovarian follicles and oocytes formation and development. Other genes with upregulated expression at this stage were enzyme arachidonate 12-lipoxygenase 12R type (*alox12b*) responsible for metabolism of a signal compound—arachidonic acid (ARA), signaling factors such as growth differentiation factor 1 (*gdf1*), Wnt11b, cell adhesion molecules claudin 6 and connexin 38, transcription factors *foxr1* and *foxh1*, and survivin—an inhibitor of apoptosis.

**Table 18:** Chosen genes upregulated in ZW versus ZZ gonads at NF62 stage [higher gene expression level in ZW than in ZZ gonads]

Probe name	Gene symbol	Gene name	Log FC
A_10_P009488	*alox12b*	Arachidonate 12-lipoxygenase, 12R	5.326002
A_10_P031553	*zp4-a*	Zona pellucida glycoprotein 4	4.350343
A_10_P032511	*cldn6.1*	Claudin 6, gene 1	4.081705
A_10_P038461	LOC398389	Survivin	3.781997
A_10_P034497	*kpna2*	Importin alpha 1b	3.634424
A_10_P048511	*foxh1*	Forkhead box H1	3.486778
A_10_P009533	*gdf1*	Growth differentiation factor 1	3.42356
A_10_P031016	*foxr1*	Forkhead box R1	3.378015
A_10_P005051	*xlzpc*	Zona pellucida C glycoprotein	2.893829
A_10_P205908	*foxh1*	Forkhead box H1	2.859015
A_10_P004066	LOC397866	Connexin 38	2.845325
A_10_P008731	*wnt11b*	Wingless-type MMTV integration site family, member 11B	2.404891

There were 2630 genes with upregulated expression in ZZ (genetic males) gonad at stage NF62 (Suppl. Table 20, and chosen genes are shown in Table 19). Functional analysis grouped these into many categories (Table 11). Among known genes with upregulated expression were factors involved in signaling and signaling pathways: *igf1*, desert hedgehog (*dhh*), sonic hedgehog (*shh*), indian hedgehog (*ihh*), *wnt3a*, *wnt8b*, *wnt7b*, Janus kinase 2 (*jak2*), frizzled receptor 4 and 10 (*fzd4*, *fzd10*), cellular retinoic acid–binding protein 2 (*crabp2*), SMAD family member 4 (*smad4*); proteases: serine protease 3 (*prss3*), cathepsin H (*ctsh*), peptidase inhibitor—serpini2; transcription factors: LIM homeobox 1 (*lhx1*), homeobox a9, d10, and d13 (*hoxa9*, *hoxd10*, *hoxd13*), *foxf1*, *foxa2*, *gata2*; extracellular matrix components: collagen III (*col3a1*), collagen I (*col1a1*), fibrillin 3 (*fbn3*); extracellular matrix enzymes: *mmp2*, *mmp16*, cell adhesion molecule 3 (*cadm3*); and intermediate filaments: keratin 15 and nestin (*nst*).

**Table 19:** Chosen genes downregulated in ZW in relation to ZZ gonads at NF62 stage [higher gene expression level in ZZ than in ZW gonads]

Probe name	Gene symbol	Gene name	Log FC
A_10_P077615	MGC116439	Uncharacterized protein MGC116439	8.36828
A_10_P045961	*prss3*	Protease, serine, 3	7.894
A_10_P075910	*serpini2*	Serpin peptidase inhibitor, clade I2	4.04603
A_10_P143593	*ctsh*	Cathepsin H	3.91699
A_10_P041916	*smad4.1*	SMAD family member 4, gene 1	3.54869
A_10_P186858	*lhx1*	LIM homeobox 1	3.43296
A_10_P067362	*igf1*	Insulin-like growth factor 1	3.21852
A_10_P037301	*dhh-b*	Desert hedgehog	3.07896
A_10_P004008	*hoxd10*	Homeobox D10	2.92652
A_10_P036201	*krt15*	Keratin 15	2.86458
A_10_P027055	*shh*	Sonic hedgehog	2.84344
A_10_P047936	*hoxd13*	Homeobox D13	2.7812
A_10_P026995	*wnt3a*	Wingless-type MMTV integration site family, member 3A	2.78056
A_10_P002038	*mmp16*	Matrix metallopeptidase 16	2.77842
A_10_P137013	*col3a1*	Collagen, type III, alpha 1	2.75957
A_10_P143748	*crabp2*	Cellular retinoic acid–binding protein 2	2.74791
A_10_P116556	*wnt8b*	Wingless-type MMTV integration site family, member 8B	2.72865
A_10_P139638	*nes*	Nestin	2.71329
A_10_P000674	*foxf1-a*	Forkhead box F1	2.69515
A_10_P232633	*fbn3*	Fibrillin 3	2.64504
A_10_P002666	*cadm3*	Cell adhesion molecule 3	2.53779
A_10_P040276	*wnt7b*	Wingless-type MMTV integration site family, member 7B	2.52685
A_10_P016774	*foxa2*	Forkhead box A2	2.48915
A_10_P050489	*jak2*	Janus kinase 2	2.48864
A_10_P000087	*fzd10-a*	Frizzled class receptor 10	2.46006
A_10_P267657	*col1a1*	Collagen, type I, alpha 1	2.43227
A_10_P162773	*gata2*	GATA binding protein 2	2.41128
A_10_P141938	*hoxa9*	Homeobox A9	2.3827
A_10_P000694	*fzd4*	Frizzled class receptor 4	2.3253
A_10_P027230	*ihh*	Indian hedgehog	2.11478
A_10_P164973	*mmp2*	Matrix metallopeptidase 2	2.05927

Genes identified here that showed sexual dimorphism of expression can be categorized into several functional groups: (1) signaling molecules: chordin (upregulated in ♀), *wnt3a* (upregulated in ♂), *wnt7b* (♂), *wnt8b* (♂), *wnt10b* (♀), *wnt11b* (♀), *igf1* (♂), *igf3* (♀ and ♂), *gdf1* (♀), *gdf3* (♂), *ccdc50* (effector in EGF pathway) (♂), including hedgehog factors (♂): *dhh*, *shh*, *ihh*; (2) retinoic binding proteins: *rbp2* (♀), *rbp4* (♀ and ♂); (3) enzymes involved in signaling: enzyme glycerophosphodiester phosphodiesterase 1 (*gde1*) responsible for synthesis of signaling molecule lysophosphatidic acid (LPA) (♂), enzyme arachidonate 12-lipoxygenase 12R type (*alox12b*) responsible for metabolism of a signal compound—arachidonic acid (♀); (4) receptors of wnt signaling: *fzd4* (♂), *fzd10* (♂); (5) proteases: cathepsin H (♂), cathepsin K (♂), calpain 8 (♀); (6) protease inhibitors: serpin A3 (♀), serpin I2 (♀ and ♂); (7) transcription factors: *foxa2* (♀), *foxf1* (♂), *foxh1* (♀), *foxo1* (♂), *foxr1* (♀), *lhx1* (♂), *lhx8* (♀), *gata2* (♂), Kruppel-like factor 9 (*klf9*) (♂), Kruppel-like factor 15 (*klf15*) (♂); (8) helicase: *ddx25* (♀); (9) cell adhesion molecules: occludin (♂), claudin 6 (♀),galectin-a (♀); (10) extracellular matrix components (mainly in ♂): collagens 1,3,9 (♂), vitronectin (♂), decorin (♂), lumican (♂), fibrillin 3 (♂); (11) extracellular matrix enzymes: *mmp1* (♀), *mmp2* (♂), *mmp7* (♀), *mmp16* (♂), *adam21* (♀), *adam27* (♀); (12) oocyte-specific proteins (♀): *zp4*, *xlzpc*; (13) epithelium-specific intermediate filaments (♂): keratins 5, 12, 14, 15.

The changes in the level of the expression of several genes listed above indicate that EGF signaling and lysophosphatidic acid (LPA) signaling may be involved in testis differentiation, arachidonic acid signaling may be involved in ovarian differentiation, while the wnt signaling, insulin-like growth factor signaling, and retinol signaling may be involved in gonad development in both sexes.

**Fig. 4:**
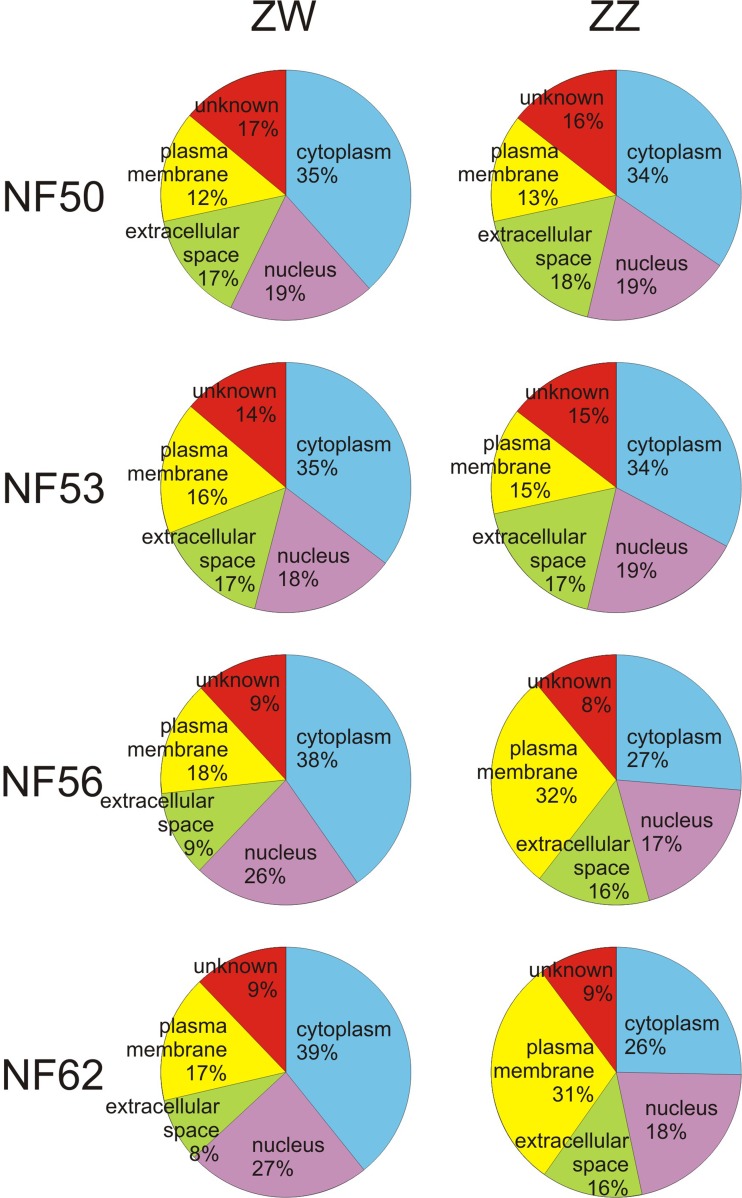
Subcellular distribution of gene products (obtained from the Ingenuity Pathway Analysis)

Interestingly, from the moment of sexual differentiation (after stage NF53), the genes encoding cytoplasmic and nuclear proteins are upregulated in ZW gonads (developing ovaries), while the genes encoding cell membrane proteins are upregulated in ZZ gonads (developing testes) (Fig. 4). The same trend was noted during gonad development in *Silurana tropicalis* (Haselman et al. 2015). This indicates that there are important molecular differences between developing ovaries and testes.

### Comparison of sex-specifically expressed genes in developing gonads of *Xenopus* and other vertebrates

We compared *Xenopus* microarray data to the published microarray data of developing gonads in other vertebrates: mouse (Jameson et al. 2012), chicken (Ayers et al. 2015), a red-eared slider *Trachemys scripta* (Czerwinski et al. 2016), American alligator (Yatsu et al. 2016)—both species with temperature-dependent sex determination, and zebrafish (Sreenivasan et al. 2008). The comparison is shown in Tables 20, 21, and 22.

**Table 20:** Comparison of sex-specifically expressed genes in developing gonads of *Xenopus* and mouse

Gene	*Xenopus laevis* (this paper)	Mouse (Jameson et al. 2012)
*Wnt3*, *Wnt7*, *Wnt8*, *Wnt10*, *Wnt11*, *chordin*	Sexual dimorphism	No sexual dimorphism
*Igf1*	Higher in ZZ	Higher in XX
*Gdf1*	Higher in ZW	Not expressed
*Igf3, Gdf3*	Higher in ZZ	Not expressed
*Ccdc50*	Higher in ZZ	No sexual dimorphism
*Dhh*, *Shh*, *Ihh*	Higher in ZZ	Only *Dhh* expressed
*Rbp*	*rbp2* higher in ZW and *rbp4* in ZZ and ZW	*Rbp1* (in XX) and *Rbp4* (in XY)
*Gde1*	Higher in ZZ	No sexual dimorphism
*Alox12b*	Higher in ZW	Not expressed
*serpins*	Several expressed	Not expressed
*Cathepsin H* (*ctsh*)	*ctsh* higher in ZZ	Only *Ctsh* higher in XY
*Foxo1*	Higher in ZZ	Higher in XY
*Lhx1*	Higher in ZZ	Higher in XY
*Col9*	Higher in ZZ	Higher in XY
*MMP2*	Higher in ZZ	Higher in XY
*calpain 8 (Capn8)*	Higher in ZW	Not expressed

**Table 21:** Comparison of sex-specifically expressed genes in developing gonads of *Xenopus* and chicken

Gene	*Xenopus laevis* (this paper)	Chicken (Ayers et al. 2015)
*calpain 5 (Capn5)*	No sexual dimorphism	Higher in ZW
*gpr56*	Not expressed	Higher in ZW
*fgfr3*	Not expressed	Higher in ZW

**Table 22:** Comparison of sex-specifically expressed genes in developing gonads of *Xenopus* and red-eared slider (*Trachemys scripta*), American alligator, and zebrafish

Gene	*Xenopus laevis* (this paper)	Red-eared slider (Czerwinski et al. 2016)	American alligator (Yatsu et al. 2016)	Zebrafish (Sreenivasan et al. 2008)
*fdxr2*	Slightly higher in ZZ	Higher at male-producing temperature	–	–
*hspb6*	Slightly higher in ZZ	Higher at female-producing temperature	–	–
*twist1*	Slightly higher in ZZ	Higher at female-producing temperature	–	–
*nov*, *pcsk6*	No sexual dimorphism	Higher at male-producing temperature	–	–
*vwa2*, *rbm20*	Not expressed	Higher at male-producing temperature	–	–
*frank1*, *avil*	Not expressed	Higher at female-producing temperature	–	–
*kdm6b*	Not expressed	Higher at male-producing temperature	–	–
*wnt11*	Higher in ZW	–	Higher at male-producing temperature	–
Estrogen receptor 2 *esr2*	Higher in ZZ	–	–	Higher in testes

The transcriptome of developing mouse gonad did not show the expression of *Wnt3*, *Wnt7*, *Wnt8*, *Wnt10*, *Wnt11*, and chordin (Jameson et al. 2012), which were expressed in *Xenopus* developing gonads. The *Igf1* was expressed in XX (genetic females) mouse gonads at a higher level than in XY gonads (Jameson et al. 2012); however, in *Xenopus*, this gene was expressed in ZZ developing gonads (genetic males). In mouse, in contrast to *Xenopus* (data presented in this study), the developing gonads did not express the *Igf3*, *Gdf1*, and *Gdf3* (Jameson et al. 2012). The *Ccdc50* was expressed in the developing mouse gonads but did not show sexual dimorphism of expression (Jameson et al. 2012). In *Xenopus*, this gene had an upregulated expression in ZZ gonads. Among hedgehog growth factors, in developing mouse gonads, only the *dhh* was expressed (Jameson et al. 2012). In *Xenopus*, gonads *dhh* and also *shh* and *ihh* were expressed. In mice, the *Rbp1* (in XX) and *Rbp4* (in XY gonads) were expressed (Jameson et al. 2012). In *Xenopus*, the *rbp2* was expressed in ZW and *rbp4* in ZZ and ZW gonads. *Gde1* gene was expressed in developing mouse gonads; however, it did not show sexual dimorphism of expression (Jameson et al. 2012) In *Xenopus*, this gene had an upregulated expression in ZZ gonads. *Alox12b* gene was not expressed in the developing mouse gonads (Jameson et al. 2012) but was upregulated in *Xenopus* ZW gonads. A subpopulation of *fzd* receptors was expressed in the developing mouse gonads. In *Xenopus*, *fzd4* and *fzd10* had an upregulated expression in developing ZZ gonads. The calpain 8 (*Capn8*) was not expressed in developing mouse gonads (Jameson et al. 2012) but was upregulated in *Xenopus* ZW gonads. The serpins were not expressed in developing mouse gonad (Jameson et al. 2012), but they were expressed in *Xenopus* developing gonads. In developing mouse gonads, several cathepsins (*Cts*) were expressed; however, only cathepsin H (*ctsh*) was upregulated in XY gonads (Jameson et al. 2012), and this gene was also upregulated in ZZ *Xenopus* gonads. Among forkhead box factors, only *Foxo1* was expressed in XY developing mouse gonads (Jameson et al. 2012) and in ZZ *Xenopus* gonads. Similarly, *Lhx1* was expressed in XY developing mouse gonads (Jameson et al. 2012) and ZZ *Xenopus* gonads. Considering proteins of extracellular matrix, only collagen 9 and metalloproteinase Mmp2 were expressed in a similar manner in XY developing mouse gonads (Jameson et al. 2012) and ZZ *Xenopus* gonads.

Analysis of transcriptome of developing chicken gonads showed that calpain 5 (*Capn5*), *Gpr56*, and *Fgfr3* were upregulated in ZW (female) gonads, which suggested that they may be involved in sexual differentiation (Ayers et al. 2015). Calpain 5 was expressed in developing *Xenopus* gonads, but not in a sex dimorphic manner. We showed the upregulation of calpain 8 in ZW (females) *Xenopus* gonads, which suggests a role of this group of proteases in sexual differentiation of vertebrate gonads. However, calpain 5 or 8 was not expressed in developing mouse gonads (Jameson et al. 2012). *Gpr56* was upregulated in XY mouse and ZW chicken gonads (Ayers et al. 2015; Jameson et al. 2012), but it was not expressed in *Xenopus* developing gonads. *Fgfr3* showed sexual dimorphism of expression in developing chicken gonads (upregulated in ZW) (Ayers et al. 2015) and was also expressed, equally in both sexes, in mouse (Jameson et al. 2012) and *Xenopus* gonads.

Analysis of transcriptome of a red-eared slider (*T. scripta*) developing gonads showed that *Vwa2*, *Fdxr*, *Nov*, *Kdm6b*, *Rbm20*, and *Pcsk6* were upregulated in the male-producing temperature, while *Fank1*, *Avil*, *Twist1*, and *Hspb6* were upregulated in the female-producing temperature (Czerwinski et al. 2016). *Fdxr2* and *Hspb6* were also upregulated in ZW (male) developing gonads of *Xenopus*, but the sexual dimorphism in the level of expression was not statistically significant. *Twist1* gene was slightly upregulated in ZZ gonads of *Xenopus*, but the sexual dimorphism in the level of expression was also not significant. We detected the expression of *Nov* and *Pcsk6* in *Xenopus* gonads but these genes did not show a sexual dimorphism of expression. Among *Kdms* genes, we detected only the expression of *kdm6a* but it did not show sexual dimorphism. We did not detect the expression of *Vwa2*, *Rbm20*, *Frank1*, or *Avil* in developing *Xenopus* gonads.

In American alligator, the expression of *Wnt11* was shown at male-producing temperature, which induces the development of the testes (Yatsu et al. 2016). We detected the expression of this gene in ZW (female) developing gonads in *Xenopus*. Analysis of transcriptome of zebrafish developing gonads showed that the estrogen receptor 2 (*esr2*) was upregulated in developing testes (Sreenivasan et al. 2008). The ZZ developing *Xenopus* gonads also upregulated the expression of this gene.

This comparison indicates that there is a profound difference in the pattern of gene expression and sexual dimorphism of gene expression between *Xenopus* and other vertebrates. Only few genes indicated above show a similar pattern of expression between *Xenopus* and other vertebrates. This shows how complex and fast-evolving is a molecular regulation of gonad development.

### Conclusion

In this study, we revealed genes representing many functional groups, which showed sexual dimorphism of expression in developing *Xenopus* gonads. Some of these genes are probably involved in sex determination and sexual differentiation of the gonads. We also detected a sexual dimorphism of expression of many uncharacterized and unnamed genes. These genes should be characterized and studied further to discover if they are involved in sex determination and sexual differentiation. Comparative analysis of genes expressed in developing gonads of different classes of vertebrates showed striking inter-specific differences. Only few genes showed similarities of expression pattern between the species. This indicates how little we know and how complex, diversified, and evolutionary malleable are molecular mechanisms driving gonad development in vertebrates.

## Material and methods

### Animals

Tadpoles of the African clawed frog (*Xenopus laevis* Daudin, 1802) were raised in 10-L aquaria (30 tadpoles per 10 L) at 22 °C, fed daily with powder food Sera Micron (Sera), and staged according to Nieuwkoop and Faber (1956). The tadpoles at four stages (NF50, NF53, NF56, and NF62) were anesthetized with 0.1% MS222 solution, and the gonads were manually dissected under the dissecting microscope. All individuals used in the experiments were handled according to Polish legal regulations concerning the scientific procedures on animals (Dz. U. nr 33, poz. 289, 2005) and with the permission from the First Local Commission for Ethics in Experiments on Animals.

### Sex determination by PCR

The genetic sex of each tadpole was determined using PCR detection of female-specific *dm-w* gene. DNA was isolated from tadpole tails using NucleoSpin Tissue Kit (Macherey-Nagel, 740952.240C). The *dm-w* gene (W-linked female-specific marker) and *dmrt1* gene (positive control) were used to determine ZZ or ZW status of tested animals. PCR was performed as previously described (Yoshimoto et al. 2008). Following pairs of primers were used: for *dm-w*, 5′-CCACACCCAGCTCATGTAAAG-3′ and 5′-GGGCAGAGTCACATATACTG-3′, and for *dmrt1*, 5′-AACAGGAGCCCAATTCTGAG-3′ and 5′-AACTGCTTGACCTCTAATGC-3′.

### Histological analysis

Bouin’s solution-fixed and paraffin-embedded samples were sectioned at 4 μm. Sections were deparafinated, rehydrated, and stained with hematoxylin and picroaniline according to Debreuill’s procedure (Piprek et al. 2012). Sections were viewed under the Nikon Eclipse E600 microscope.

### RNA isolation

Total RNA was isolated using Trizol and purified with Direct-zol RNA kit according to the manufacturer’s protocol (Zymo Research, R2061). The total RNA was quantified using NanoDrop 2000, and RIN (RNA Integrity Number) was assessed with Bioanalyzer 2100. All samples used in the study had RIN above 8. In order to obtain a sufficient amount of RNA, the samples from 10 individuals were pooled in each experiment as previously described (Piprek et al. 2018). Total RNA in RNase-free water was frozen at − 80 °C until further use.

### Microarray analysis

Microarray analysis was performed as previously described (Piprek et al. 2018). Total RNA was labeled with fluorescent dyes using Agilent One-Color Quick Amp Labeling Protocol. RNA isolated from ZW gonads were labeled with Cy3, and RNA from ZZ gonads with Cy5. Fluorescently labeled RNA samples were mixed with Agilent Hi-RPM Hybridization Buffer, and hybridized at 65 °C for 17 h in HybArray12 hybridization station (Perkin Elmer). RNA from ZW and ZZ were mixed together and hybridized to the same chip. The RNA isolated from the gonads in different stages of development was labeled with the same fluorochrome (either Cy3 or Cy5) and hybridized individually to the separate chips. Samples were washed in Gene Expression Wash Buffer 1 (6X SSPE, 0.005% N-lauroylsarcosine; at RT) and Gene Expression Wash Buffer (0.06X SSPE, 0.005% N-lauroylsarcosine; at RT) for 1 min each and immersed in a solution of acetonitrile. Air-dried slides (custom-commercial Agilent-070330 *X. laevis* Microarray slides) were scanned in the Agilent Technologies G2505C Microarray Scanner at a 5-μm resolution. The microarray experiment was repeated three times.

### Data processing

Data processing was performed as previously described (Piprek et al. 2018). TIF files obtained in microarray scanner were processed using Agilent Feature Extraction software version 10.5.1.1. Control and non-uniform features were removed; remaining values for each unique probe sequence were averaged. Log base 2 intensities were median centered between arrays. Differential gene expression was filtered using a statistical significance threshold (FDR < 0.05) and a fold change threshold (2-fold). The data were published in Gene Expression Omnibus (accession number GSE105103). Functional analysis and gene ontology were carried out using DAVID 6.8 (https://david.ncifcrf.gov/tools.jsp) and IPA (Ingenuity Pathway Analysis, Qiagen). First, we compared the level of gene expression between gonads in different stages of development within each sex. The gene expression level at each stage of gonad development was compared to the gene expression level at the previous developmental stage, i.e., the stage NF53 was compared to the stage NF50, the stage NF56 was compared to the stage NF53, and the stage NF62 was compared to the stage NF56. In each comparison, the level of gene expression in the younger stage of gonad development was arbitrarily designated as the reference level of expression. The results of these analyses gave us an overview of the pattern of gene expression in consecutive stages of gonad development. Subsequently, we compared the level of gene expression between genetic female (ZW) versus male (ZZ) gonads at each studied developmental stage.

## Electronic supplementary material

ESM 1 (XLSX 38 kb)

ESM 2 (XLSX 88 kb)

ESM 3 (XLSX 19 kb)

ESM 4 (XLSX 19 kb)

ESM 5 (XLSX 83 kb)

ESM 6 (XLSX 151 kb)

ESM 7 (XLSX 60 kb)

ESM 8 (XLSX 45 kb)

ESM 9 (XLSX 36 kb)

ESM 10 (XLSX 35 kb)

ESM 11 (XLSX 33 kb)

ESM 12 (XLSX 74 kb)

ESM 13 (XLSX 69 kb)

ESM 14 (XLSX 38 kb)

ESM 15 (XLSX 25 kb)

ESM 16 (XLSX 76 kb)

ESM 17 (XLSX 15 kb)

ESM 18 (XLSX 34 kb)

ESM 19 (XLSX 58 kb)

ESM 20 (XLSX 182 kb)
